# Retraction Note: LncRNA HOTAIR regulates HIF-1α/AXL signaling through inhibition of miR-217 in renal cell carcinoma

**DOI:** 10.1038/s41419-024-07111-9

**Published:** 2024-10-01

**Authors:** Quan Hong, Ou Li, Wei Zheng, Wen-zhen Xiao, Lu Zhang, Di Wu, Guang-yan Cai, John Cijiang He, Xiang-mei Chen

**Affiliations:** 1 Department of Nephrology, Chinese PLA General Hospital, Chinese PLA Institute of Nephrology, State Key Laboratory of Kidney Diseases, National Clinical Research Center for Kidney Diseases, Fuxing Road 28, Haidian District, Beijing, 100853 China; 2 Division of Nephrology, Department of Medicine, Icahn School of Medicine at Mount Sinai, New York, NY 10029 USA

Retraction Note to: *Cell Death and Disease* 10.1038/cddis.2017.181, published online 11 May 2017

The Editors-in-Chief have retracted this article because, after the publication of the article, concerns have been raised regarding the image overlap of Figure 5b (si-NC) and (si-HOTAIR) with Figure 2d (Ki-67 +MVs) and (Ki-67 + Control) of [1] respectively. The authors stated that the experiments of ki-67 staining of the renal cell carcinoma in this article were outsourced to a third-party laboratory. Due to the age of the article, the authors were unable to provide the raw images. The Editors-in-Chief therefore no longer have confidence in the results and conclusions presented in this article.

None of the authors have responded to any correspondence from the editor/publisher about this retraction.
